# Delayed Biliary Hemorrhage due to Pseudoaneurysm Rupture Caused by Migration of Placed Plastic Stent After Endoscopic Ultrasound‐Guided Hepaticogastrostomy

**DOI:** 10.1002/deo2.70238

**Published:** 2025-11-04

**Authors:** Yu Akazawa, Masahiro Ohtani, Yosuke Murata, Takuto Nosaka, Tomoko Tanaka, Kazuto Takahashi, Tatsushi Naito, Yasunari Nakamoto

**Affiliations:** ^1^ Department of Gastroenterology, Faculty of Medical Sciences University of Fukui Fukui Japan

**Keywords:** biliary hemorrhage, endoscopic ultrasound‐guided hepaticogastrostomy, plastic stent migration, pseudoaneurysm, transcatheter arterial embolization

## Abstract

Endoscopic ultrasound‐guided hepaticogastrostomy (EUS‐HGS) is an effective method for cases where transpapillary approaches to pancreato‐biliary diseases are challenging, though serious complications often occur. Here, we report an extremely rare case of delayed biliary hemorrhage due to pseudoaneurysm rupture after EUS‐HGS, caused by migration of the placed plastic stent. The patient was pathologically diagnosed with unresectable advanced pancreatic cancer and presented with severe duodenal stenosis and bile duct obstruction. Before chemotherapy, EUS‐HGS with a biliary plastic stent (7Fr Type IT stent) was successfully performed without early complications. However, after 46 days, the patient developed massive melena, and computed tomography revealed a biliary hemorrhage within the common bile duct. Imaging revealed that the hepatic end of the plastic stent had migrated from the hepatic hilum to the posterior segment. After 71 days, the patient experienced a recurrent biliary hemorrhage, and an 8 mm pseudoaneurysm was identified in the posterior hepatic region at a location consistent with the migrated hepatic end of the plastic stent. Hemostasis was successfully achieved by emergency transcatheter arterial embolization with N‐butyl cyanoacrylate. During the 6 months after the intervention, no recurrence of the pseudoaneurysm was observed, and the patient continued systemic chemotherapy with stable disease control. We suggest that biliary hemorrhage due to pseudoaneurysm rupture, which may be caused by migration of the placed plastic stent, should be considered a life‐threatening late complication of EUS‐HGS, requiring thorough follow‐up.

## 1 INTRODUCTION

Hemobilia is a relatively uncommon condition with the potential for fatal outcomes. Iatrogenic factors are frequently implicated in its development. Hemobilia resulting from pseudoaneurysm formation following self‐expandable metallic stent (SEMS) insertion into the bile duct has been often reported [1]. However, pseudoaneurysm formation after plastic stent placement is extremely rare.

Recently, endoscopic ultrasound‐guided hepaticogastrostomy (EUS‐HGS) has gained widespread acceptance as an effective intervention for patients with hepatobiliary diseases for whom transpapillary biliary drainage is challenging [2, 3, 4, 5]. Although this procedure offers less invasiveness compared to traditional therapeutic options, including surgical and percutaneous approaches, it can be frequently associated with serious complications [3, 4]. In particular, bleeding mostly occurs immediately after EUS‐HGS, and late‐onset hemorrhage occurring from several days to months after EUS‐HGS is uncommon [4].

Here, we report an extremely rare case of delayed biliary hemorrhage due to pseudoaneurysm rupture caused by the migration of a plastic stent placed after EUS‐HGS. This highlights the importance of considering potential late‐onset pseudoaneurysm rupture when gastrointestinal bleeding occurs after biliary plastic stent placement using EUS‐HGS.

## 2 CASE REPORT

A 75‐year‐old woman with persistent vomiting was admitted to the University of Fukui Hospital. Contrast‐enhanced computed tomography (CT) performed on admission revealed a hypovascular lesion in the pancreatic head and para‐aortic lymph node enlargement, leading to severe duodenal stenosis and biliary obstruction. Subsequently, EUS‐guided fine‐needle biopsy (EUS‐FNB) of the pancreatic lesion was performed, and pathological analysis confirmed the definitive diagnosis of pancreatic ductal adenocarcinoma, cStage IV (T3N0M1), according to the Union for International Cancer Control TNM Classification of Malignant Tumors, 8th edition.

**FIGURE 1:**
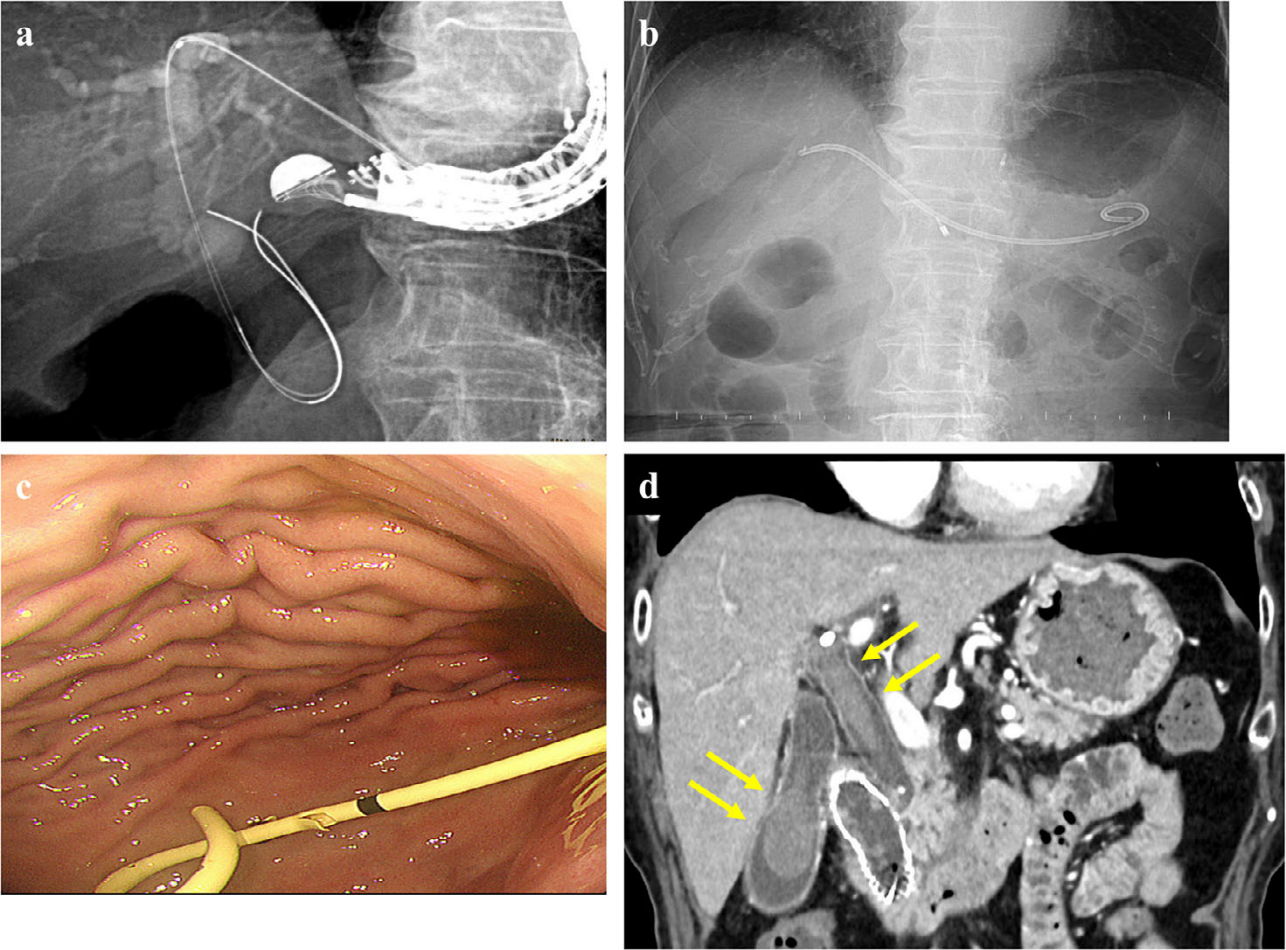
X‐ray and endoscopic images of endoscopic ultrasound‐guided hepaticogastrostomy (EUS‐HGS) performed before chemotherapy administration and Contrast‐enhanced tomography images of biliary bleeding on day 46 after EUS‐HGS. (a, b) A 7‐Fr plastic stent for EUS‐HGS (Type IT Stent) was placed with the hepatic side end of the plastic stent at the hepatic hilum via B3. (c) The stomach end of the plastic stent was placed at the lesser curvature of the lower body of the stomach, and bile flowed out through the plastic stent. (d) On day 46 after EUS‐HGS, Biliary bleeding, which was visualized as a high‐density area, was observed in the common bile duct and gallbladder (yellow arrow).

EUS‐HGS was performed before chemotherapy induction because the transpapillary approach is difficult due to severe duodenal stenosis. Using an EUS scope (EG‐580UT; Fujifilm Corporation, Tokyo, Japan), the B3 bile duct was punctured from the posterior wall of the midgastric body using a 19‐G needle (EZ Shot 3; Olympus Corporation, Tokyo, Japan). After inserting a 0.025‐inch guidewire (Visiglide2; Olympus Corporation, Tokyo, Japan), the fistula was dilated using a 7‐Fr drill dilator (Tornus ES; Asahi Intec Co., Ltd., Aichi, Japan), and we subsequently performed additional dilation using a 4‐mm balloon dilator (REN; Kaneka Medix, Tokyo, Japan) because the tract could not be sufficiently dilated with the drill dilator alone, ensuring safe and smooth stent insertion. Subsequently, a 7‐Fr plastic stent (7‐Fr 14cm Type IT plastic stent; Gadelius Medical Co., Ltd., Tokyo, Japan) was placed, with the hepatic end of the stent at the hepatic hilum due to the biliary anatomy (Figure 1a–c). Concurrently, an uncovered duodenal metallic stent (22 × 120 mm Niti‐S Stent; Taewoong Medical Co., Ltd., Gimpo‐si, Gyeonggi‐do, South Korea) was placed in the descending duodenum. These endoscopic interventions were successfully performed without any early complications, and 7 days after EUS‐HGS, gemcitabine and nab‐paclitaxel (GnP) therapy were initiated as the first‐line chemotherapy.

**FIGURE 2:**
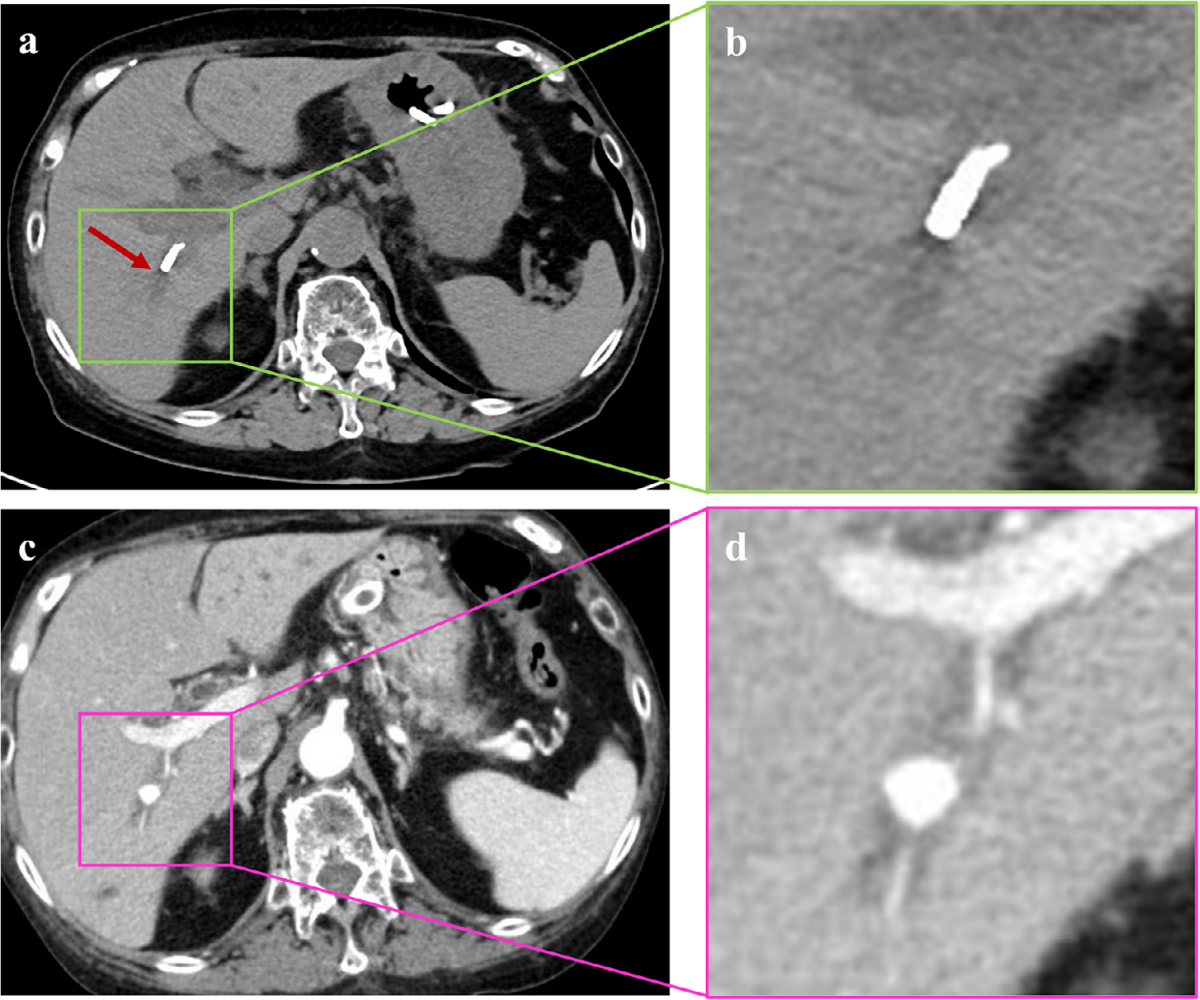
Computed tomography (CT) images on day 46 and day 71 after EUS‐guided hepaticogastrostomy (EUS‐HGS). (a, b) A CT image on 46 days after EUS‐HGS. The hepatic end of the plastic stent had spontaneously migrated and was located in the posterior area of the liver (red arrow). (c), (d) A CT image on 71 days after EUS‐HGS. An 8 mm pseudoaneurysm was observed in the posterior area of the liver, which is a location consistent with the hepatic end of the plastic stent.

However, 46 days after EUS‐HGS, the patient experienced sudden melena and was admitted for emergency hospitalization at the University of Fukui Hospital. Laboratory findings at the time of hospitalization revealed anemia (hemoglobin, 7.8 g/dL) and jaundice (total bilirubin, 3.5 mg/dL). Furthermore, contrast‐enhanced CT revealed high‐attenuation areas within the common bile duct and gallbladder, leading to a diagnosis of bile hemorrhage (Figure 1d). Notably, the hepatic end of the 7‐Fr plastic stent, which had initially been placed at the hepatic hilum after EUS‐HGS, had migrated approximately 2 cm into the posterior segment of the liver (Figure S1 and Figure 2a,b). We removed the previously placed 7‐Fr plastic stent and replaced it with an 8 mm × 12 cm partially covered self‐expandable metallic stent (Niti‐S S‐type Spring Stopper Stent; Taewoong Medical Co., Ltd., Gimpo‐si, Gyeonggi‐do, South Korea). Subsequently, no further melena was observed after admission, and the source of bleeding was unidentifiable.

**FIGURE 3:**
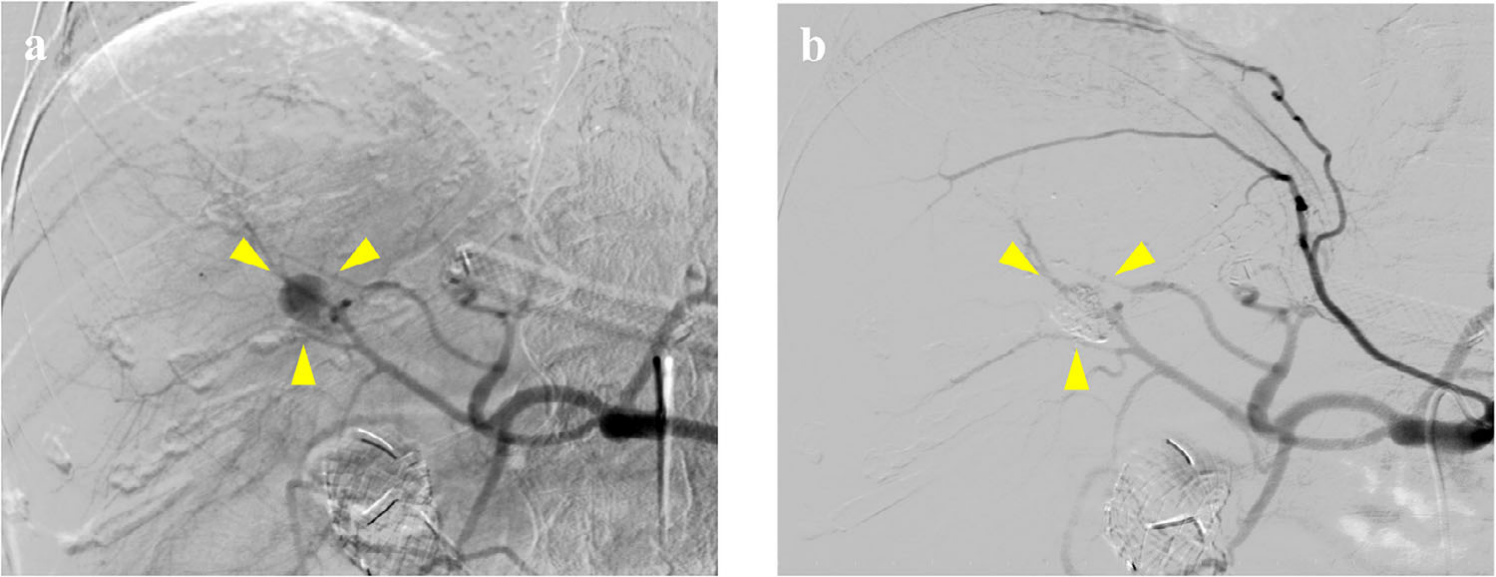
Urgent abdominal angiography. (a) A pseudoaneurysm is observed at the posterior segmental arteries of the liver (yellow arrowhead). (b) Transcatheter arterial embolization was performed using an NBCA‐Lipiodol mixture, and the blood flow within the aneurysm disappeared.

Seventy‐one days after EUS‐HGS, the patient presented with massive melena recurrence, severe anemia (hemoglobin 7.0 g/dL), and jaundice (total bilirubin 3.8 mg/dL). Contrast‐enhanced CT revealed a hemorrhage within the common bile duct, and an 8 mm pseudoaneurysm was identified in the posterior segmental branch of the hepatic artery, which was perfectly consistent with the position of the hepatic end of the migrated plastic stent (Figure 2c,d). Biliary hemorrhage due to pseudoaneurysm rupture was diagnosed, and emergency transcatheter arterial embolization (TAE) of the aneurysm was immediately performed. A microcatheter was inserted into the right femoral artery, and angiography revealed a pseudoaneurysm in the posterior segmental hepatic artery branch. Embolization was performed with an *n*‐butyl cyanoacrylate (NBCA)–lipiodol mixture (0.5mL, NBCA:Lipiodol = 1:3), leading to complete blood flow occlusion to the pseudoaneurysm (Figure 3a,b).

The patient's general condition improved rapidly, and she was discharged 15 days after an episode of biliary hemorrhage. Approximately 6 months after TAE, no recurrence of the pseudoaneurysm was observed. Furthermore, GnP therapy was continued, and effective control of the tumor was maintained.

## 3 DISCUSSION

EUS‐HGS has recently been established as an effective drainage method for malignant biliary obstructions. A meta‐analysis reported that EUS‐HGS achieved a high technical success rate and contributed significantly to symptom improvement [2]. However, EUS‐HGS is effective; nevertheless, serious complications may occur. Hemorrhage is a characteristic complication with an adverse event rate of 18.2% [3]. In this case, the initial bleeding episode was difficult to identify; however, prompt management of the rebleeding event contributed to successful hemostasis and prevented critical outcomes.

Biliary hemorrhage is relatively rare and can be fatal, particularly when iatrogenic. The formation of pseudoaneurysms is recognized as one of the serious complications of endoscopic retrograde cholangiopancreatography (ERCP). Reportedly, the incidence of pseudoaneurysms after metallic stent placement using ERCP is 1.2% [1], and the time for pseudoaneurysm formation after transpapillary metallic stent placement ranges from 5 days to 2 years [6]. Delayed hemorrhage due to pseudoaneurysm after EUS‐HGS, as observed in this case, is extremely rare. To the best of our knowledge, only five previous cases have been reported (Table 1) [7, 8, 9], all involving metallic stents.

**TABLE 1:** Overall results of cases of pseudoaneurysm formation associated with endoscopic ultrasound‐guided hepaticogastrostomy (EUS‐HGS).

Author, year	Age/ Sex^a^	Symptoms	Disease	Stent	Size of aneurysm	Affected artery	Time from EUS‐HGS to rupture	Treatment	Rebleeding
Prachayakul et al., 2013 [7]	66/M	Melena	Pancreatic cancer	10 mm × 10 cm SEMS	—	LHA	14 days	TAE	None
Hoshi et al., 2023	78/F	Hematemesis	Pancreatic cancer	8 mm × 12 cm SEMS	—	LHA	7 days	TAE	None
Yamada et al.,2023 [8]	65/M	Fever	Pancreatic cancer	8 mm × 10 cm SEMS	6 mm	LHA	50 days	TAE	None
Suzuki et al., 2024	93/M	Hematemesis	Stone	8 mm × 12 cm SEMS	5 mm	LHA	59 days	TAE	None
Ban et al., 2025 [9]	80/F	Hematemesis	Pancreatic cancer	10 mm × 7 cm SEMS	10 mm	LHA	60 days	TAE	None
Our case	75/F	Melena	Pancreatic cancer	7Fr × 14 cm Plastic Stent	8 mm	RHAp	46 days	TAE	None

^a^ Sex. M, male; F, female.

Abbreviations: EUS‐HGS, endoscopic ultrasound‐guided hepaticogastrostomy; LHA, left hepatic artery; RHAp, right hepatic artery posterior; SEMS, self‐expandable metal stent; TAE, transcatheter arterial embolization.

Pseudoaneurysm formation after biliary metallic stent placement results from the strong radial/axial forces of stents and metallic friction, which weakens the vascular wall [10]. In this case, a pseudoaneurysm developed despite the placement of a plastic stent that exerted far weaker mechanical forces. We hypothesized that the substantial migration of the liver‐side end of the stent from the hepatic hilum to the posterior segment of the liver contributes to pseudoaneurysm formation by irritating the posterior segmental hepatic artery branch, leading to vascular damage. Furthermore, Slight stent movement caused by diaphragmatic motion may have irritated the artery and contributed to pseudoaneurysm formation. Also, localized inflammation, including cholangitis, might promote vascular wall fragility. In addition, chemotherapy with gemcitabine and nab‐paclitaxel may have contributed to pseudoaneurysm formation by causing mucosal injury and inflammation, leading to vascular fragility. We suggest that pseudoaneurysm formation may have been prevented by appropriate stent positioning or by using a shorter stent. However, a short stent may compress the left biliary tree and increase the risk of left hepatic artery pseudoaneurysm. Deeper placement of the IT stent in the common bile duct might have reduced this risk.

Active bleeding due to pseudoaneurysm rupture is life‐threatening and leads to rapid hemodynamic deterioration, requiring immediate and reliable hemostasis. In this case, emergency TAE was performed for the ruptured pseudoaneurysm. The successful outcome of this case reveals that prompt management is critical for improving the prognosis of patients with delayed hemorrhage due to pseudoaneurysms after EUS‐HGS.

In conclusion, we experienced an extremely rare case of delayed biliary hemorrhage due to pseudoaneurysm rupture that resulted from the migration of a plastic stent placed after EUS‐HGS. We suggest that it is important to consider the possibility of pseudoaneurysm formation originating from biliary plastic stents in patients with gastrointestinal bleeding after EUS‐HGS.

## Author Contributions

**Yu Akazawa**, **Masahiro Ohtani**, and **Yasunari Nakamoto**. designed the study. **Yu Akazawa** collected and analyzed the data. **Yu Akazawa** wrote the manuscript. All the authors have reviewed and approved the final manuscript.

## Conflicts of Interest

The authors declare no conflicts of interest.

## Funding

This case report was partially supported by Japan Society for the Promotion of Science (JSPS) KAKENHI Grant‐in‐Aid for Scientific Research Number 25K19290. The funders had no role in study design, data collection and analysis, decision to publish, or preparation of the manuscript.

## Ethics Statement

**Approval of the research protocol by an Institutional Review Board**: N/A

## Consent

Informed consent was obtained from all patients for inclusion in the study.

## Supporting information




**FIGURE S1**: A Computed tomography (CT) image on day 1 after EUS‐guided hepaticogastrostomy (EUS‐HGS). The liver end of the plastic stent was located at the hepatic hilum (yellow arrow).
