# Supplementary figures and images for: Delayed Biliary Hemorrhage due to Pseudoaneurysm Rupture Caused by Migration of Placed Plastic Stent After Endoscopic Ultrasound‐Guided Hepaticogastrostomy
Source: DEN Open. 2025 Nov 4;6(1):e70238. doi: 10.1002/deo2.70238 (PMC12584874; doi:10.1002/deo2.70238)

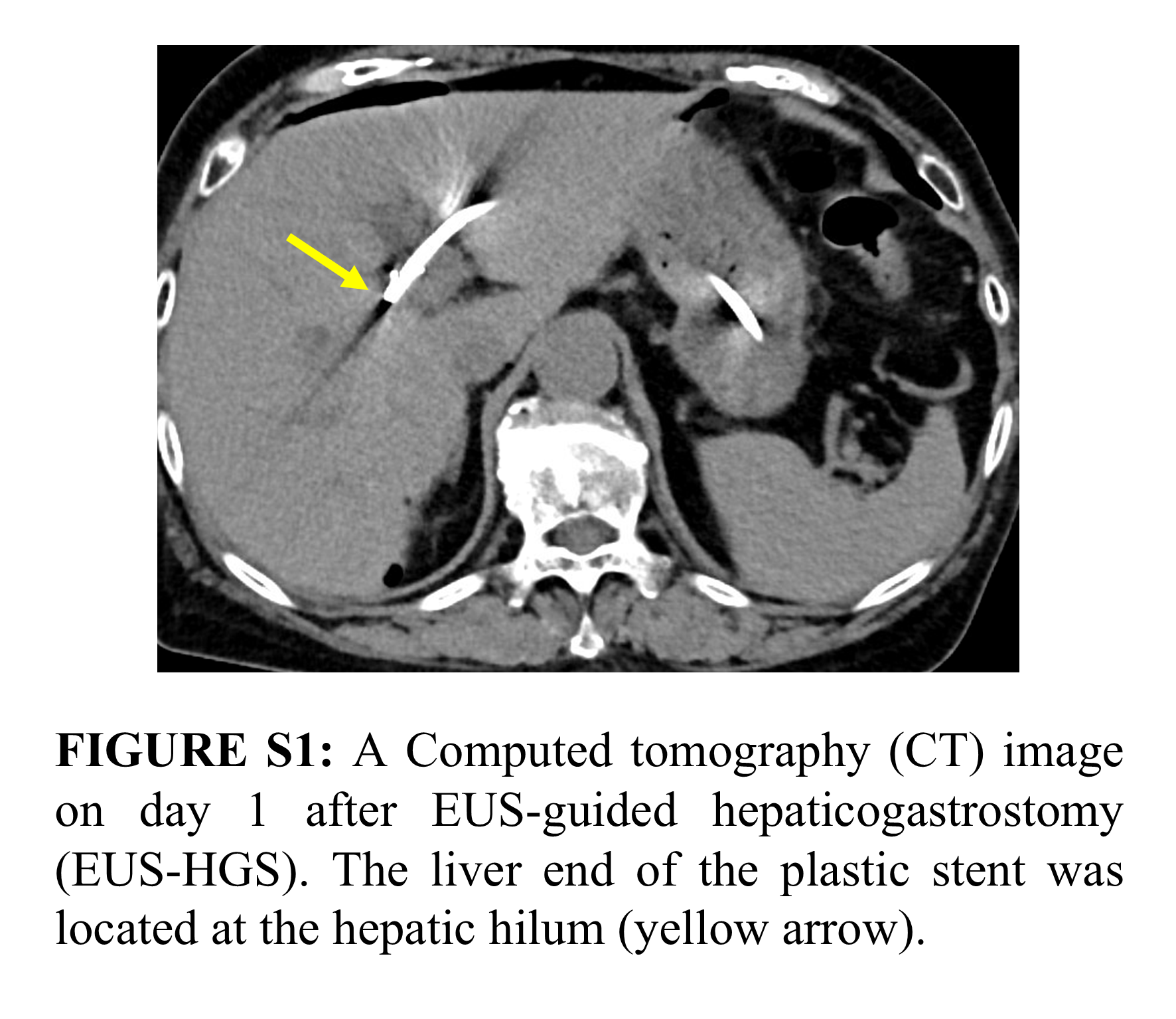

Supplement: Supplementary file 1 — FIGURE S1: A Computed tomography (CT) image on day 1 after EUS‐guided hepaticogastrostomy (EUS‐HGS). The liver end of the plastic stent was located at the hepatic hilum (yellow arrow). [file DEO2-6-e70238-s001.tif]
